# A study protocol: using demand-side financing to meet the birth spacing needs of the underserved in Punjab Province in Pakistan

**DOI:** 10.1186/1742-4755-11-39

**Published:** 2014-05-30

**Authors:** Syed Khurram Azmat, Moazzam Ali, Waqas Hameed, Ghulam Mustafa, Ghazanfer Abbas, Muhammad Ishaque, Mohsina Bilgrami, Marleen Temmerman

**Affiliations:** 1 Research, Monitoring & Evaluation Department, Marie Stopes Society, Karachi, Pakistan; 2 Department of Reproductive Health and Research, World Health Organization, Geneva, Switzerland; 3 Department of Uro-gynecology, University of Ghent, Ghent, Belgium

**Keywords:** Demand-side financing, Voucher, Social franchising, Quasi-experimental, Maternal health, Unmet need for modern contraception, Birth spacing, Study protocol, Punjab, Pakistan

## Abstract

**Background:**

High fertility rates, unwanted pregnancies, low modern contraceptive prevalence and a huge unmet need for contraception adversely affect women’s health in Pakistan and this problem is compounded by limited access to reliable information and quality services regarding birth spacing especially in rural and underserved areas. This paper presents a study protocol that describes an evaluation of a demand-side financing (DSF) voucher approach which aims to increase the uptake of modern contraception among women of the lowest two wealth quintiles in Punjab Province, Pakistan.

**Methods/Design:**

This study will use quasi-experimental design with control arm and be implemented in: six government clinics from the Population Welfare Department; 24 social franchise facilities branded as *‘Suraj’* (Sun), led by Marie Stopes Society (a local non-governmental organization); and 12 private sector clinics in Chakwal, Mianwali and Bhakkar districts. The study respondents will be interviewed at baseline and endline subject to voluntary acceptance and medical eligibility. In addition, health service data will record each client visit during the study period.

**Discussion:**

The study will examine the impact of vouchers in terms of increasing the uptake of modern contraception by engaging private and public sector service providers (mid-level and medical doctors). If found effective, this approach can be a viable solution to satisfying the current demand and meeting the unmet need for contraception, particularly among the poorest socio-economic group.

## Background

Stagnating indicators for several reproductive and child health conditions in many countries across Africa and Asia are a major concern for national governments and development partners that are striving to achieve the Millennium Development Goals (MDGs) [1]. The progress being made on MDG 4 (to reduce child mortality) and MDG 5 (to improve maternal health) is in line with projections but continues to be far too slow, especially for MDG 5 [2]. Family planning is one of the most effective interventions in terms of costs and promoting health. It has the potential to reduce poverty and hunger and prevent around 30% of maternal deaths and 10% of child deaths [3]. Without contraceptive use, maternal deaths are projected to be 1.8 times higher [4]. With this in mind, international donors are becoming increasingly interested in linking the financing of health services to the achievement of results, rather than to inputs [5].

In response to developing countries’ health-related needs, the concept of demand-side financing (DSF) was developed to improve access to and use of health services, particularly among the poor. DSF is seen as a tool that can improve the quality of care and use of under-utilized services among needy and underserved populations by placing purchasing power, as well as the choice of provider (where possible), directly in the hands of the recipients [6,7]. Demand-side financing/voucher programme typically engage the private sector, ensuring minimum quality standards to accredit facilities and encourage providers who do not qualify to make improvements to become eligible [8]. This introduces greater competition among providers, thereby increasing supply and improving consumers’ choice [9]. Performance-based financing (PBF) – or ‘pay-4-performance’ or ‘output-based aid (OBA)’ – generally consists of a range of methods and approaches aimed at linking incentives to performance [10]. They have been used in many countries [11,12], and are gaining increasing attention as a way of achieving national goals in low-income countries [10,13].

The World Health Organization (WHO) emphasizes the need to set up partnerships with private practitioners to remedy these problems, using a range of methods, including social franchising [14]. The franchising of sexual and reproductive health services has recorded successes in family planning programming in Asia, Africa and Latin America and provides a possible solution to meeting growing demands for health care from the public [15,16]. In developing countries, franchising has also emerged as an important strategy for provider training by private sector organization.

However, there has still been limited research on the effectiveness of DSF in both high- and low-income countries [17]. Significant weaknesses in the current evidence base on the success of performance-based payment initiatives were also found [12]. Many questions remain unanswered or unevaluated due to the lack of controls and the interference of confounding factors [12]. There is a need for more rigorous research that elicits the true impact of performance-based financing [18]. This involves gathering rigorous evidence from well-designed studies on the effectiveness of DSF in lower-income countries generally and specifically for family planning services.

### Context

In 1950, Pakistan had a population of 37 million and was the world’s 13th largest country, as measured by population. By 2009, Pakistan had become the sixth largest country in the world, with 180 million people [19]. The Total Fertility Rate (TFR) in Pakistan is 3.8 children per woman [20] and is 6.6 among married women of reproductive age (MWRA) [21]. One in every three pregnancies is unplanned [21]. Among currently married women aged 15–49, 96% know of at least one method of family planning [21]. However, use of modern contraception is low at just 26.1% [20]. Among modern contraceptives, female sterilisation and condom are the most common contraceptive choice in Pakistan [20]. Use of highly effective long-acting and reversible methods (such as intrauterine devices – IUDs – and implants) is negligible [20].

The median age of marriage in Pakistan for a woman is 20 years old in urban areas and 19 years old in rural areas, while the median age of a woman at the time of her first pregnancy is 22 years old. There is a clear and huge demand for family planning in Pakistan [20], despite the fact that contraceptive prevalence rate (CPR) increased by 20% between 1990 to 2010 from 12.2% to 32.4% for all methods and the unmet need for family planning reduced by around 4% from 30.2% to 25.9% during the same period [22].

Moreover, health-care policies in Pakistan have traditionally focused on public financing and provision through supply-side subsidies. Low-priced or officially ‘free’ public health care is intended to ensure the entire population has equitable access to care. However, most people, including many poor people, seek better quality health care in the private sector and are forced to pay out of their own pockets, owing to the absence of quality public health services [23].

Meeting the unmet need for contraception could have the immediate effect of reducing Pakistan’s TFR to three births per woman, thereby putting the country on the trajectory for reaching replacement fertility in the near future [21]. If only 4% of current oral contraceptive users (100,000 women) in Pakistan switched to IUDs or implants, it is estimated that more than 25,000 unintended pregnancies could be averted over a five-year period [24].

Marie Stopes Society (MSS), a local non-governmental organization, piloted its own version of a social franchise model in 2008. Today, this includes 130 *Suraj* social franchise partners, integrated with a DSF voucher scheme, as well as an enterprising cadre of 130 field health educator (FHE) in 21 districts across the country [25-28]. The model is essentially a partnership between MSS and private sector local health services providers. It aims to increase demand, access, choices and the quality of family planning services for rural, underserved and poor communities, similar to a ‘social franchise model’. These providers belonged to and were practising in rural, poor and underserved areas. *Suraj* providers were trained and accredited to provide condoms, emergency contraceptives, injectable, oral contraceptives and to insert and remove IUDs. The model has significantly stimulated consumer demand and has addressed unmet need for family planning in the intervention areas [27,28]. Additionally, it was documented that social franchising used alongside free vouchers for long-term contraceptive choices significantly increased awareness and use of modern contraception [27,28].

### Research question

Does the demand-side financing (DSF) voucher approach increase the uptake of modern contraception^a^ among women of the lowest two wealth quintiles in Punjab Province, Pakistan?

## Objectives

To evaluate the effectiveness of the DSF voucher approach in increasing the uptake of modern contraception among women from the lowest two wealth quintiles in Punjab Province, Pakistan

*In order to evaluate the effectiveness at population level, service delivery level as well as to describe the efficiency and process issues of the intervention approach, the following table elaborates a detailed list of key indicators and the sources of their data (refer to* Table 1*).*

**Table 1:** List of key indicators

**Dimension addressed**	**Variable/indicator**	**Data source**
**Effectiveness at the population level**	1. Contraceptive prevalence rate among women living in lowest two wealth quintiles	Population-based household survey in project areas
	2. % of clients recommending services to others	Endline population-based household survey; voucher validation
**Effectiveness at the service delivery level**	3. Number of new FP acceptors *(disaggregated by long-term versus short-term FP)*	Routine service delivery records
	4. Number of clients who never used FP before receiving services from study providers	Routine service delivery records
	5. Number and% of post-partum (PP) clients accepting an FP method (disaggregated by long-term versus short-term FP)	Routine service delivery records
	6. % of vouchers validated	External voucher validation survey
	7. Number and% of service providers adhering to medical/clinical standards	Clinical quality assurance monitoring
	8. % of family planning services provided through vouchers (disaggregated by FP method)	Routine service data
	9. % of referrals made by FHE for family planning services	Routine service data
**Efficiency and process issues**	*Prospects for sustainability*	Financial records
	10. Monthly service income	
	*Cost effectiveness*	Financial records; routine service delivery records and registers
	11. Cost per couple year protection (CYP)	
	12. Cost per new FP acceptor	
	*Provider attrition/turnover*	Baseline and endline service provider assessment
	13. % of study providers offering FP services at the start of the study who are still participating at the end of the project	
	*Training*	Baseline and endline service provider audit
	14. % of providers trained by MSS on different dimensions of FP who are providing services for which they were trained (disaggregated by type of service, e.g. IUD insertion, PAC FP)	

## Methods/Design

### Intervention

This operations research proposal is designed to implement and demonstrate the utility of the DSF approach devised with slight modifications to the original MSS *Suraj* social franchise model (described above). Table 2 details the components of the intervention arm:

**Table 2:** Details of intervention components

**S.no**	**Intervention components**	**Description**
1	Training on reproductive health/family planning and post-training evaluation	**Medical:** Reproductive health and family planning, counselling, quality of services, infection prevention, short-term family planning methods, IUD & Femplant insertion and removal.
		**Business:** Basic budgeting skills, record keeping, stock management, branding, marketing and the voucher management. The training is followed by post-training evaluation conducted by external consultant (medical doctor).
2	Field health educator (FHE)	Field educators are local community members; they undergo training on family planning methods, voucher distribution systems and data recording. They pay door-to-door visits, raise awareness, generate referrals, conduct follow-up visits and distribute vouchers for the short-term and long-term methods to eligible women, identified through poverty scale.
3	Branding (only for intervention 1 group: *Suraj*)	Mid-level providers in intervention areas are branded as *Suraj* while marketing is done through FHE, posters, wall paintings, leaflets, etc. The *Suraj* logo is displayed prominently in Urdu outside all clinics.
4	Voucher for short-term and long-term contraceptive method	Vouchers are for short-term and long-term methods, and are distributed by FHE to eligible women, identified through poverty scale. They can be redeemed at any project provider. Later, the reimbursement is sent to the provider against her claim. Femplant cannot be inserted by mid-level providers as per the national health policy. Femplant clients from *Suraj* areas will be referred to nearby public or urban private providers.

The overall design of the intervention is the same. However, there is a small variation in terms of component specifications and the geographical location of service providers. We will work with three different cadres of service providers (see Table 3):

**Table 3:** Description of intervention and comparison arms

**Description**	**Intervention group 1**	**Intervention group 2**	**Intervention group 3**	**Comparison group**
	**Rural private providers (*Suraj *** **Model)**	**Urban public providers**	**Urban private providers**	**Mix of mid-level, public and urban providers**
**Location**	Rural	Peri-urban	Urban	Rural, peri-urban and urban
**Type of provider**	Mid-level private providers (e.g. physicians, nurses, midwives, lady health visitors (LHVs))	Public providers (e.g. basic health unit (BHU), population welfare department (PWD) doctors	Private providers (e.g. doctors)	Private and public doctors and mid-level providers
**S.no**	**Intervention components**			
**1**	Selection, training and accreditation	Selection, training, and post-training evaluation	Selection, training, and post-training evaluation	Selection, training and post-training evaluation
**2**	Branding, marketing: IEC material, wall chalking etc.	-	-	-
**3**	Field worker mobilisation	Field worker mobilisation	Field worker mobilisation	-
**4**	Free vouchers short-term and long-term (IUD only*) methods	Free vouchers short- term and long-term methods	Free vouchers short-term and long-term methods	-

*According to Pakistan health policy, only qualified doctors can insert Femplant.

• Group 1 will be an MSS *Suraj* social franchise model targeting the rural population with a cadre of mid-level private-sector service providers (e.g. nurse, midwife, female health visitor).

• Group 2 will include private-sector medical doctors (minimum criteria to have at least Bachelor of Medicine and Bachelor of Surgery (MBBS) that will be located in the urban areas.

• Group 3 will include public-sector medical doctors working at public health facilities (e.g. Basic Health Unit, Population welfare department, or Rural Health Centre etc.) located in the peri-urban areas of the selected districts.

• A mixture of the above-mentioned providers will be selected for the comparison group.

All cadres of service providers will receive the same medical training regarding short- and long-term family planning methods. The only exception will be Femplant/implant insertion and removal training, which will not be given to mid-level (Group 1) providers because, following the national health policy, they are not allowed to practice this surgical procedure. An FHE, a local community resident, will be assigned to each service provider to create awareness about family planning and its availability at the respective service provider centre.

In addition, the important component of the proposed intervention is the free vouchers for all family planning services except the permanent methods (male and female sterilization). These vouchers will be distributed by FHE in their catchment population following a standard poverty assessment. However, Femplant/implant services will not be provided by the Group 1 service providers (who are mid-level providers). However, the women in the respective catchment areas will be given the option of Femplant vouchers and that voucher can be redeemed at the nearest service provider centers’ (Group 2 or 3).

Lastly, mid-level providers in Group 1 will be branded as *Suraj* while FHE will also be carrying out marketing activities including posters, wall paintings/chalking and direction boards, etc. The *Suraj* logo will be displayed prominently in Urdu (the local language) outside all clinics.

The service providers in the comparison arm, by contrast, will only receive the same medical training and will not be exposed to FHE, vouchers or marketing/branding. However, they will share statistics regarding routine services on a quarterly basis for data analysis purposes. It should be noted that these communities are not ‘pure’ controls in the sense that, as they will be exposed to training and due to the nature of maternal & neonatal health (MNH) and reproductive health (RH) programming in Pakistan, there may be a number of other entities—governmental and non-governmental—engaged in these activities. We will take every care to select communities that are not only comparable with the intervention communities on aspects other than the intervention, but where other family planning (FP) and RH activities are limited. Trend analysis of routine service statistics (e.g. number of new FP adopters and first-time FP users) will enable us to ascertain whether favourable outcomes would have occurred even in the absence of the three intervention models that are being tested.

### Study design

We will use a pre- and post- quasi-experimental study design with a comparison arm. The main features of this methodology include:

a. the use of intervention and comparison groups (without random assignment, hence quasi-experimental);

b. study outcomes will be assessed at the start of programme implementation, as well as 24 months after the start of intervention;

c. quantitative techniques and analytical methods will be used – data collection methods will include a population-based household survey, providers’ assessment, and special qualitative studies during the course of intervention, routine services, external quality audit and voucher validation survey, and monitoring (clinical quality assessment) data.

### Study settings

We will conduct this research in three districts of Punjab Province, which are selected based on key socio-demographic and reproductive health indicators (see Table 4). Mianwali and Chakwal will be assigned to intervention groups, while Bhakkar will serve as a comparison group. Within these districts, we will identify health facilities based on set criteria. All service providers in each district, whether intervention or comparison arm, will have its own defined catchment area – which is around 25,000 for intervention Group 1 service provider and 35–40,000 for intervention Groups 2 and 3.

**Table 4:** Comparability of intervention and comparison districts

**S.no**	**Key indicators**	**Selected districts**			**Punjab province**
		**Chakwal**	**Mianwali**	**Bhakkar**	
1	Est. district population 2011 (in thousands)	1376	1342	1335	
2	% of pop. who are female age 15-49	25	23	22.0	22.3
3	Contraceptive prevalence rate (combined)	28	20	20	32
4	% of modern contraceptive user	23	16	17.3	25.1
5	% of traditional contraceptive user	5.5	3.7	2.7	7.1
6	% of women who are literate	56.7	42.8	51.3	46.6
7	Unemployment rate	12.4	4.9	6.7	6.8
8	Infant mortality	60	78	82	77
9	% of household with electricity	94	86	90.0	92.5

### Study population

Married women of reproductive age (MWRA) are a common target group for family planning interventions. However, since the project aims to test the effectiveness of vouchers, women living in the lowest two wealth quintiles will also be specifically targeted for the provision of family planning services through vouchers.

### Demand-side population

• Women aged 15–49 years (including but not limited to recently delivered/post-partum women)

• Post-abortion care (PAC) clients

• Post-partum family planning clients.

### Supply-side population

The above groups reflect populations in which MSS would like to observe key outcomes. However, the supply-side target groups (i.e. the groups that will serve as implementers of the family planning interventions) are:

• 24 (16 in intervention and 4 in comparison arm) mid-level private-sector providers (branded as *Suraj*) based in rural areas.

• 12 (8 in intervention and 4 in comparison arm) private sector medical doctors based in urban areas

• 6 (4 in intervention and 2 in comparison arm) public-sector medical doctors based in peri-urban areas.

### Proposed data collection procedure

#### ***Population-based survey***

The survey will be conducted in the surrounding catchment of 28 selected health-care facilities (16 located in rural and 12 in urban) in intervention arm, and 14 (8 in rural and 6 in urban) facilities in comparison arm. The total population around each health-care facility, located in rural areas, ranges from 20,000-25,000; whereas each peri-/urban facility covers a population of around 35,000-40,000.

A total of 1,276 interviews will be carried out in the intervention arm and, to maintain statistical power, 2,552 women will be interviewed in the comparison arm, which is almost twice the sample of the intervention arm. Multi-stage sampling techniques will be used for the selection of survey participants within both the study arms. In the first stage, purposive selection of the district will be made, followed by the selection of the catchment area where the programme is to be in operation, then the households, and finally the eligible respondent will be selected. Prior to data collection, all the households (within a 4–7 kilometer radius) around each selected health-care facility will independently be allotted a unique identifier. The sampling within each study arm will be done using probability proportional to size sampling methods, whereby a larger sample with more households will be obtained around the health-care facility. Epi-info version 6.04 [29] will be used for the random selection of households. Lastly, within the selected households, the youngest eligible woman will be invited to participate in the survey. The baseline was conducted in May 2012 whereas the end of project survey will take place in July 2014. Face-to-face interviews will be carried out using a semi-structured questionnaire used in Pakistan Demographic Health Survey of 2006–07. Local enumerators will be hired and trained to use the questionnaire and will be trained on ethics for data collection. Study investigators will monitor and supervise the entire activity. The WHO will lead the evaluation survey towards the end of the study by hiring an independent research firm.

#### ***Sample size calculation for population-based surveys***

The hypothetical assumption is that the modern contraceptive prevalence rate will increase by up to 20 percentage points from the baseline to the endline survey in the intervention area and that, in the comparison area, it will increase by 5 percentage-points between baseline and endline survey. Using PASS 11 software, group sample sizes of 1,276 and 1,276 will produce a two-sided 95% confidence interval for the difference in population proportions with a width that is equal to 0.05. However, due to three different versions of intervention being tested under this study, the sample of comparison sites will be doubled to maintain study power.

### Eligibility

To be eligible, a woman must a) be of reproductive age (15–49 years), b) be married, c) residence within the geographical area specified for each clinic, and d) voluntarily provides informed consent.

#### ***Pre- and post-assessment of service providers***

Service provider assessment shall be carried out during the identification process before providers are exposed to any intervention such as training. The assessment is broadly divided into two parts:

a) *Service* provider, which includes documentation of detailed profile, their experience, knowledge in terms of the trainings received, current practices with regard to service provision, and client flow;

b) Facility assessment will include observation of facility infrastructure, availability of necessary equipment and waste disposal management.

The assessment will be carried out on all selected service providers in both the intervention and comparison arms. At the time of the interview/assessment, all the ethical guidelines will be followed, including informing them about the benefits and risks of their participation and informed consent, voluntary participation and confidentiality of information.

#### ***Routine services statistics***

In order to measure the effectiveness at the service delivery level, routine service statistics shall be captured from each service provider through a specially designed daily data record diary/register. These registers will capture client-level information such as age, number of children, services received, new and continuing clients, voucher and non-voucher clients, post-partum family planning client etc. Each provider will be trained on data recording. Separate data recording sheets will be developed for comparison arm providers where they will only record the cumulative number of services at the end of the day and they will share the data on a quarterly basis. However, we will review the data recording a register of public providers and gather the data for each project health facility from the district-level health management information system (HMIS). The services data will be used for multiple purposes, particularly to measure trends in the number of family planning services using time series charts.

#### ***External voucher validation survey***

In order to ensure transparency, to understand disbursement and redemption dynamics, and to strengthen voucher distribution, an external voucher validation survey will be conducted every year. An external research firm will be selected following a competitive bidding process. This survey will be conducted in all intervention areas where the voucher clients will be randomly selected using a stratified probability proportion to size (PPS) technique. Each voucher will have a unique code that will be entered into an Excel database once claimed for reimbursement by service providers. Using computer software, these unique codes will be randomly selected from the database by service providers.

### Tools for data collection

All data collection tools (see Table 5) for study participants will be developed by the Principal Investigator (PI) and MSS Research, Monitoring and Evaluation Team. The tools will be pre-tested and will be available in both English and Urdu.

**Table 5:** Data collection tools, its description and evidence to be derived

**S.no**	**Tool**	**Description**	**Evidence on**
1	• Structured questionnaire for married women of reproductive age 15–49 (household survey)	Small-sample household survey with married women of reproductive age (15–49) (conducted at baseline and endline)	Population-level effectiveness
2	• Service provider assessment tool	1. Records and registers maintained by FP providers in order to generate routine monitoring by FP providers; routine service delivery statistics	Service-level effectiveness
	• Daily register for routine tracking by service providers		
	• Monthly reporting format (to be submitted by service providers to MSS)	2. Government of Pakistan routine statistics (e.g. provider-level HMIS data)	
3	• External voucher validation survey	1. Voucher validation reports	Efficiency and process issues
	Data quality assurance checklists (to be used during routine field supervision visits)	2. Financial records (from private *Suraj* providers, public and urban providers)	
		3. Service provider assessment (conducted at baseline and endline)	
		4. Field monitoring officers (will use monitoring checklist)	

### Schedule and description of research activities

The following Table 6 shows a concise breakdown of research and monitoring activities in support of the project’s objectives.

**Table 6:** Research activity schedule and description

**Activity phase**	**Duration (in months)**	**Activity details**
**Inception**	0-3	Identification of private providers; Service provider assessment (baseline); Recruitment and training of project field staff; Field-testing and finalisation of all research and monitoring tools developed for the project
**Baseline**	4-5	Small-sample population-based household survey
**Service provision & routine monitoring of programme activities**	5-31	Provision of family planning services; routine collection, prospective client follow-up, reporting and use of service delivery statistics; data quality assessments; external voucher validation survey (each year); and special qualitative studies with clients, providers and project staff.
**Endline**	31-34	Small-sample household survey; Service provider assessment (endline); collection/verification of financial data for the cost-effectiveness analysis; data entry and cleaning.
**In-depth analysis**	34-36	In-depth comparative analysis of baseline and endline data; triangulation with qualitative special studies and services data
**Knowledge dissemination**	35-36	Research reports; peer-reviewed publications; flyers; briefs; presentation at different forums; other topical dissemination products

### Data management

All the data (except for routine services data, which will be entered only once) will be double entered into pre-designed software by two separate data operators. Measures will be taken to ensure the quality of collected data. All the forms will be checked on a daily basis for completeness, logical errors, and unclear or irrelevant responses. Monitoring visits will also be made by the PI, co-investigators and project head office team to ensure the quality of data and adherence to the study protocol. If needed, refresher training will be arranged to emphasise understanding of data collection tools. The software is also restricted for mandatory fields and extreme values. Hard copies of completed tools (questionnaires, checklists, reporting formats) will be kept in a locked storage file before and after data entry, analysis and report writing. The storage file will be kept in a secure location within office premises. Only authorised personnel will have access to the locked cabinet.

### Voucher management

Each voucher will have a printed unique code with a watermark and brand symbols to ensure its uniqueness. When distributing vouchers, FHE will record information about the vouchers in a daily dairy. The data includes service information (for which the voucher is being provided), client contact details and identifiers that will be used for further validation purposes. Field supervisors will hold weekly meetings with FHEs and pay scheduled and unscheduled visits to check the information collected on vouchers and daily registers. Moreover, 20% of the vouchers claimed for reimbursement will be validated by respective district-level field supervisors. Vouchers will then be checked for completeness by district in-charge staff, head office operational staff, and finally by the finance department. Reimbursement will not be released unless all mandatory fields are properly filled out and random selected cases are validated by field supervisors.

### Data entry and analysis packages

Quantitative data will be entered into an electronic database using Visual FoxPro 6.0 and EpiData 3.1; and for analysis, we will use Stata version 13.0 and Statistical Package for Social Scientists (SPSS) version 22.0.

### Data analysis

We will limit analysis of routine service data to descriptive statistics such as frequencies, proportions, percentage distributions and cross-tabulations. Trends over time will be examined. By virtue of the quasi-experimental nature of our study with a comparison arm, it has a limited non-random assignment of individuals to comparison and intervention groups. Therefore, to isolate the effect of the intervention, we will calculate difference-in-differences estimates using multilevel random intercept logistic regression. We will use the conventional cut-point of p <0.05 as the criterion for establishing the statistical significance of findings.

## Discussion

This quasi-experimental study will investigate the impact of vouchers in terms of increasing the uptake of modern contraception through engaging private and public sector service providers (mid-level and medical doctors). We believe this study, if it is effective, will offer a viable solution for satisfying the current demand and meeting the unmet need for modern contraception – particularly among the poorest socio-economic group – by working with private as well as public sector service providers to integrate DSF. The project will illustrate implementation of a DSF – together with its evaluation findings – for client-centred, quality FP/RH services that can be adopted for replication/scale up in the public and private health sector in Pakistan, as well as in other countries.

The findings from this study will be widely shared with stakeholders, including donors, technical collaborative partners (such as WHO), government and the non-governmental organization sector through reports and seminars at the national level. We will also use the study findings to contribute to the knowledge base through peer-reviewed publication in an international journal and presentation at relevant international and regional conferences. The findings will also be presented at both family planning and forums and conferences on health systems (including health-care financing). Through publication in peer-reviewed journals, we will ensure that the experience is available in the public domain for policy-makers, implementers and other end-users and researchers. Finally, we will advocate for the use of results from this study with MSS advocacy work and the work of its partners with government partners in Pakistan.

### Technical support

The role of the Department of Reproductive Health and Research (RHR) at WHO is to provide technical advisory support to the in-country project team/Principal Investigator. This is not just limited to supervising the Monitoring and Evaluation (M&E) aspect of the project, but also to reviewing and providing continuous feedback on the project documents, as well as conducting annual field visits to the study sites and creating an enabling environment for informed decision-making on operational issues, research and policy or strategy. In summary, the RHR will provide technical support to all four project components including project design; field work and guidelines; survey and analysis and information, education and communication (IEC). The RHR role extends from strengthening the capacity of the local research project team by providing direct/indirect support and agreement on the conceptualisation of the study design; refinement and approving the study hypotheses and proposal; ensuring the harmonisation of key indicators, including the survey tool development and approval; standardisation of sampling and data collection procedures; and selection (of both Marie Stopes Society (MSS)/Marie Stopes International (MSI)-Greenstar Social Marketing (GS)/Population Services International (PSI) projects); preparation and submission of relevant ethics applications to the national bioethics committee; ensuring that the informed consent forms conform to national and international ethics standards; and reviewing the data analysis plan and the report. In addition, the RHR will also review the provider interview survey forms; routine services data collection tools; voucher management information statistics and franchise management information statistics. The RHR Department will also collaborate with the PI to determine the most appropriate way of conducting the special qualitative studies and develop the endline survey and comparative evaluation design. Moreover, multiple peer-reviewed publications in professional international journals, best practices, case studies, conference presentations and policy briefs will be produced through a consultative process that involves the RHR technical adviser and the study PI/project team.

### Ethical considerations

Maintaining high ethical standards in research and practice is paramount. We are committed to ensuring that this research proposal and the actual implementation of the proposed research project adhere to core standards in research ethics. Following the national and international ethics standards, informed consent forms have been developed and translated into Urdu (the native language). Every respondent, irrespective of whether information is being elicited using quantitative methods, will be informed about the nature and objectives of the project, any real or perceived benefits or risks, and assurances of confidentiality. Part of the informed consent process will entail reminding the respondent that s/he has the right to refuse to answer any question posed by the interviewer, and that s/he may also stop the interview at any time. The respondent will then be required to provide consent before participating in the study. The informed consent process will be conducted in a manner that is appropriate for the local language, literacy level and comprehension level of the target population. There will be no financial incentives for participation in the study. Moreover, no personal identifying information will be recorded on questionnaires that can be used to link recorded information to a specific individual. Steps will also be taken to ensure privacy during interviews. Designated field workers/supervisors will control access to hard-copy forms and questionnaires before submission to the designated research members. We are committed to ensuring that individual freedom of choice regarding the use of FP, as well as the specific FP method, is maintained throughout the project. Even for aspects of the intervention models that address financial barriers to access (voucher scheme as a component of all versions of intervention models), we will ensure that a range of FP options are available to all clients for the entire continuum of care (from community mobilisation and marketing activities to point of care with a FP service provider).

The study protocol has received approval by Research Ethics Committee of National Bioethics Committee (Ref: No. 4-87/12/NBC-92/RDC/3548). Written informed consent will be taken from all survey participants.

### Potential risks

Participation in this study has no potential risks to the participants except that loss of confidentiality may occur during the study. However, all study staff will be trained to ensure that they will protect the confidentiality of participants to the fullest extent possible. Participants’ contact information and informed consent documents with participants’ names will be stored separately in lockers. No personal identifiers for participants will appear on any data or documentation sent to the funding organization. Participants will not be identified by name in any report or publication resulting from the study data.

### Potential benefits

There will be no financial incentives for participation in the study. However, the proposed operations research study will directly benefit underserved urban and rural women in the two intervention districts. The study defines ‘underserved’ women as those who are unable to: access quality services, pay for services, make informed reproductive health choices to space their pregnancies; MWRA, post-partum women, those in need of post-abortion contraceptive, those who have never used any contraception, those who are living with unmet need for family planning, and women facing socio-cultural resistance/barriers and financial challenges to receiving FP services. They will be especially targeted and served. Vouchers redeemable for quality FP services will be made available to women, especially those in the two lowest wealth quintiles, by a FHE that will empower underserved women to decide/choose from the range of available services and providers.

In addition, 28 service providers (24 private – 16 rural and 8 urban, and 4 public) will benefit from the project as their capacities/skills to provide FP services will be enhanced and sustained through training, regular monitoring for compliance, promptly addressing challenges in meeting medical quality assurance protocols/standards, and ensuring providers’ access to commodities/supplies. Along with 28 service providers in the intervention districts, 14 public and private service providers in the comparison district will also directly benefit from capacity building training regarding FP service provision. We are confident that providers will benefit from an increase in walk-in clients to their facilities, in addition to those seeking redemption of vouchers provided by the FHE for services. The cadre of 28 FHE will gain from the project in the long term through their enhanced skills in interpersonal communications, FP counselling, side effect management and follow up.

### Limitations

The Hawthorne Effect, described as the phenomenon in which subjects in behavioral studies change their performance in response to being observed, is a possible effect that could be observed over time through prospective evidence creation. This effect could be reflected from the perceptions of private providers and female community workers regarding the intervention effectiveness. Therefore, we will take several measures to minimize this by conducting evaluation activities through an external consultant and informing participants in detail before any data collection about the study’s purpose and confidentiality elements. During regular monitoring from the research team, some random visits will be made to check the reliability of data.

## Endnotes

^a^Modern contraceptive methods: Pill, Condom, IUCD, Injectable, Female Sterilisation, Male Sterilisation, Implants.

## Competing interests

The authors, though, are affiliated with the implementing organization however they neither come under nor are not part of program team. The study was conceptualized and conducted by them independently without any consultation with the implementing field team.

## Authors’ contributions

SKA & WH was involved in conception and design of the study, analysis and interpretation of the literature, and prepared the draft; GM, MA, SKA, WH, GA and MI added literature and reviewed the analyzed content; MT and MA contributed in revising it critically for substantial intellectual content; MI, WH, GA, GM, MA and SKA supervised the data collection, data cleaning and initial analysis. All authors read and approved the final manuscript.
